# To eat or not to eat: a critical review on the role of autophagy in prostate carcinogenesis and prostate cancer therapeutics

**DOI:** 10.3389/fphar.2024.1419806

**Published:** 2024-06-07

**Authors:** Natalie Jayne Kurganovs, Nikolai Engedal

**Affiliations:** Autophagy in Cancer Lab, Institute for Cancer Research, Department of Tumor Biology, Oslo University Hospital, Oslo, Norway

**Keywords:** autophagy, prostate, cancer, carcinogenesis, therapy, androgen deprivation, radiation, chemotherapy

## Abstract

Around 1 in 7 men will be diagnosed with prostate cancer during their lifetime. Many strides have been made in the understanding and treatment of this malignancy over the years, however, despite this; treatment resistance and disease progression remain major clinical concerns. Recent evidence indicate that autophagy can affect cancer formation, progression, and therapeutic resistance. Autophagy is an evolutionarily conserved process that can remove unnecessary or dysfunctional components of the cell as a response to metabolic or environmental stress. Due to the emerging importance of autophagy in cancer, targeting autophagy should be considered as a potential option in disease management. In this review, along with exploring the advances made on understanding the role of autophagy in prostate carcinogenesis and therapeutics, we will critically consider the conflicting evidence observed in the literature and suggest how to obtain stronger experimental evidence, as the application of current findings in clinical practice is presently not viable.

## 1 Prostate cancer and current aspects of prostate cancer therapy

**FIGURE 1:**
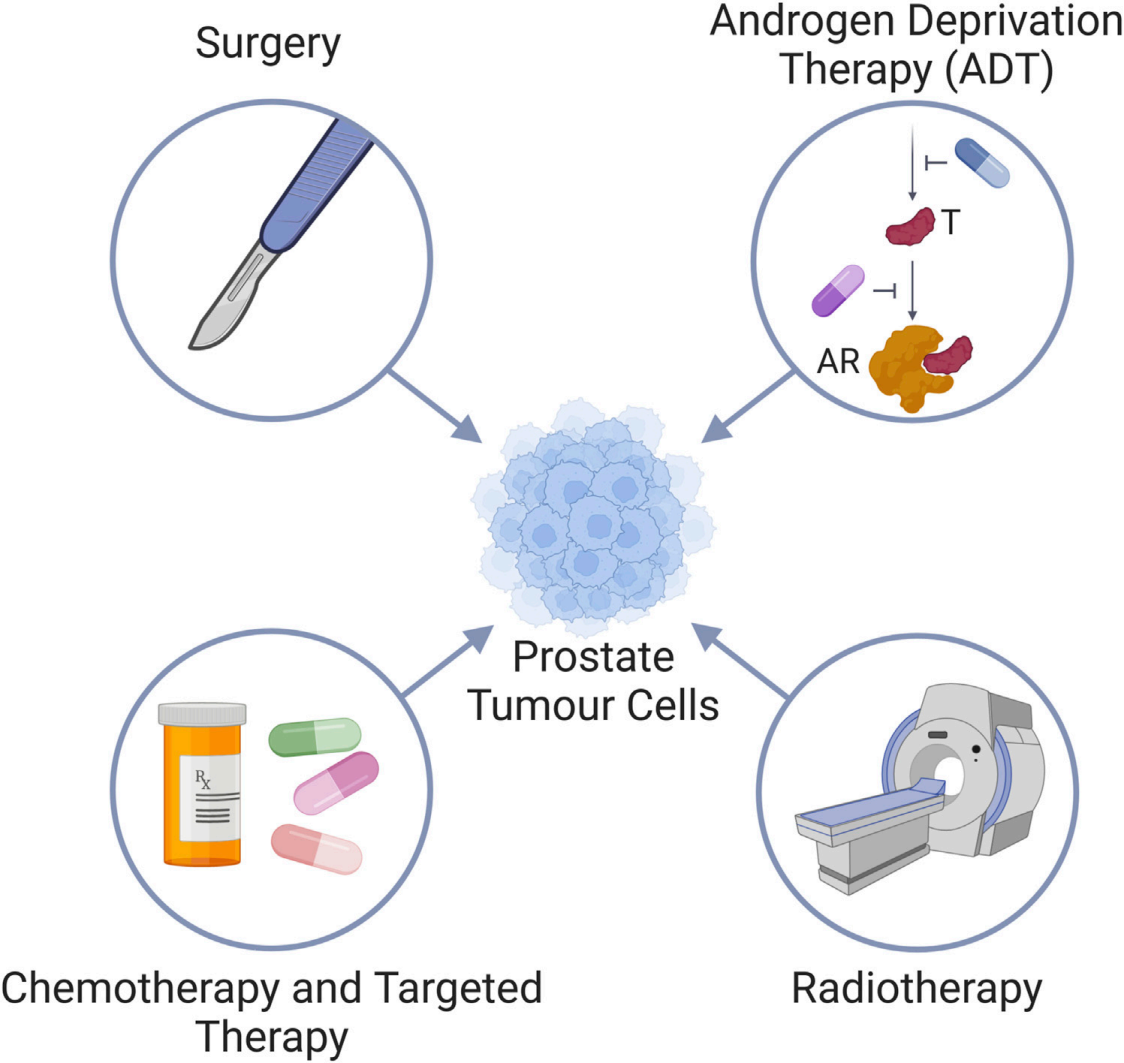
Standard treatment modalities for prostate cancer. Prostate cancer is treated via (i) surgery, (ii) Androgen deprivation therapy (ADT) by either inhibiting the androgen synthesis pathway (blue pill) to reduce testosterone (T) levels, or use of androgen receptor (AR) antagonists (purple pill); (iii) radiotherapy, and (iv) chemotherapy and targeted therapies.

The prostate is the most cancer-prone internal organ in men and is the highest cause of cancer-associated mortalities in Western countries (Siegel et al., 2023). The survival rate of patients diagnosed with prostate cancer (commonly abbreviated as PCa) is dependent on the clinical stage. Whilst early stage and confined disease may be managed by surgery, more advanced stages of prostate cancer require additional therapy, and frequently becomes very challenging to treat (Moul, 2004). The non-surgical standard of care for advanced prostate cancer is androgen deprivation therapy (ADT) (Parker et al., 2020), due to the pivotal role the androgen receptor (AR) plays in prostate cancer progression (Ferro et al., 2021), and radiation therapy (Parker et al., 2020), which causes cell death through either direct or indirect nuclear DNA damage via the generation of free radicals (Zou et al., 2017; Chaiswing et al., 2018) (Figure 1). While both ADT and radiation are administered with curative intent for men with localised prostate cancer, treatment resistance remains a serious clinical concern. Around 70% of patients treated with ADT show indications of disease progression within 2 years, despite the therapy resulting in exceptionally low levels of circulating testosterone (Crawford et al., 1989; Eisenberger et al., 1998; Mottet et al., 2011; James et al., 2015). 25%–50% of high-risk prostate cancer patients treated with radiation therapy develop biochemical recurrence within 5 years following treatment, with up to 30% succumbing to their disease within 10 years (Zietman et al., 2005; Agarwal et al., 2008; Boorjian et al., 2011). Following the development of treatment resistance, or metastatic lesions, chemotherapies and targeted therapies are also utilized in prostate cancer patients. Chemotherapies are systemic medications that inhibit cell proliferation and/or promote cancer cell death. First identified to have an effect on prostate cancer in the 1990s, chemotherapies are currently advised for use in both hormone-sensitive and -insensitive metastatic prostate cancer (Tannock et al., 1996; Tannock et al., 2004; de Bono et al., 2010; Eisenberger et al., 2017). Alas, resistance to chemotherapy occurs frequently and only half of the patients respond to docetaxel, which is the current mainstay of chemotherapy for prostate cancer patients. As a result, docetaxel only improves median survival by 2 months (Petrylak et al., 2004; Tannock et al., 2004). Thus, more efficient therapies are needed. Newer chemotherapeutics are utilised as second line therapies, following the initial failure of a first line such as docetaxel. These include mitoxantrone and cabazitaxel, which can extend median overall survival just over a year (de Bono et al., 2010). More recently, targeted therapies such as Poly-ADP ribose polymerase inhibitors (PARPi) have become a promising therapeutic option for metastatic prostate cancer (Pezaro, 2020; Risdon et al., 2021; Taylor et al., 2023) due to the induction of synthetic lethality in cells with homologous recombination repair deficiency (HRD) (de Bono et al., 2020; de Bono et al., 2021; Smith et al., 2022). HRD is found in approximately 30% of metastatic prostate cancer patients (Mateo et al., 2015; Robinson et al., 2015). PARPi can also be employed in combination with ADT (Hussain et al., 2018). However, in spite of very encouraging clinical effects, the development of treatment resistance is a major challenge also with PARPi therapies (Rose et al., 2020).

The mechanisms controlling the development and persistence of treatment resistance in prostate cancer remain poorly characterised, and filling this knowledge gap is imperative to improve prostate cancer therapeutic options. There have been many indications in the literature that autophagy may play a key role in carcinogenesis (cancer formation and progression) and treatment resistance. Below, we will discuss the available data concerning the role of autophagy in prostate carcinogenesis and prostate cancer therapeutics.

## 2 Autophagy

Autophagy (meaning “self-eating”) is a vital homeostatic process required for essential functioning of the cell and organism, and can affect various cellular activities. It is an evolutionarily conserved process that was first discovered in the 1950s and 60s through the emergence of electron microscopy (Novikoff and Essner, 1962; De Duve and Wattiaux, 1966). In this mechanism, redundant or dysfunctional cellular components are recycled in a lysosomal-dependent manner (Xie and Klionsky, 2007; Ohsumi, 2014; Trelford and Di Guglielmo, 2021). Autophagy can be classed as either *bulk autophagy* (largely non-selective degradation and recycling of bulk cytoplasm) or *selective autophagy* (targeted recycling of organelles, protein aggregates, or other cellular components).

There are three main types of autophagy; chaperone-mediated autophagy, microautophagy, and macroautophagy. Chaperone-mediated autophagy refers to a type of selective autophagy that identifies individual proteins for unfolding and direct import to the lysosome via a protein complex involving LAMP2A (Tekirdag and Cuervo, 2018). Microautophagy is the least characterised out of the three subtypes and involves lysosomal or endosomal uptake of cytoplasmic contents through direct invagination of the lysosomal or endosomal (in which case the process is termed endosomal microautophagy) limiting membrane (Sahu et al., 2011; Li et al., 2012; Schuck, 2020). The final and main type of autophagy is known as macroautophagy.

**FIGURE 2:**
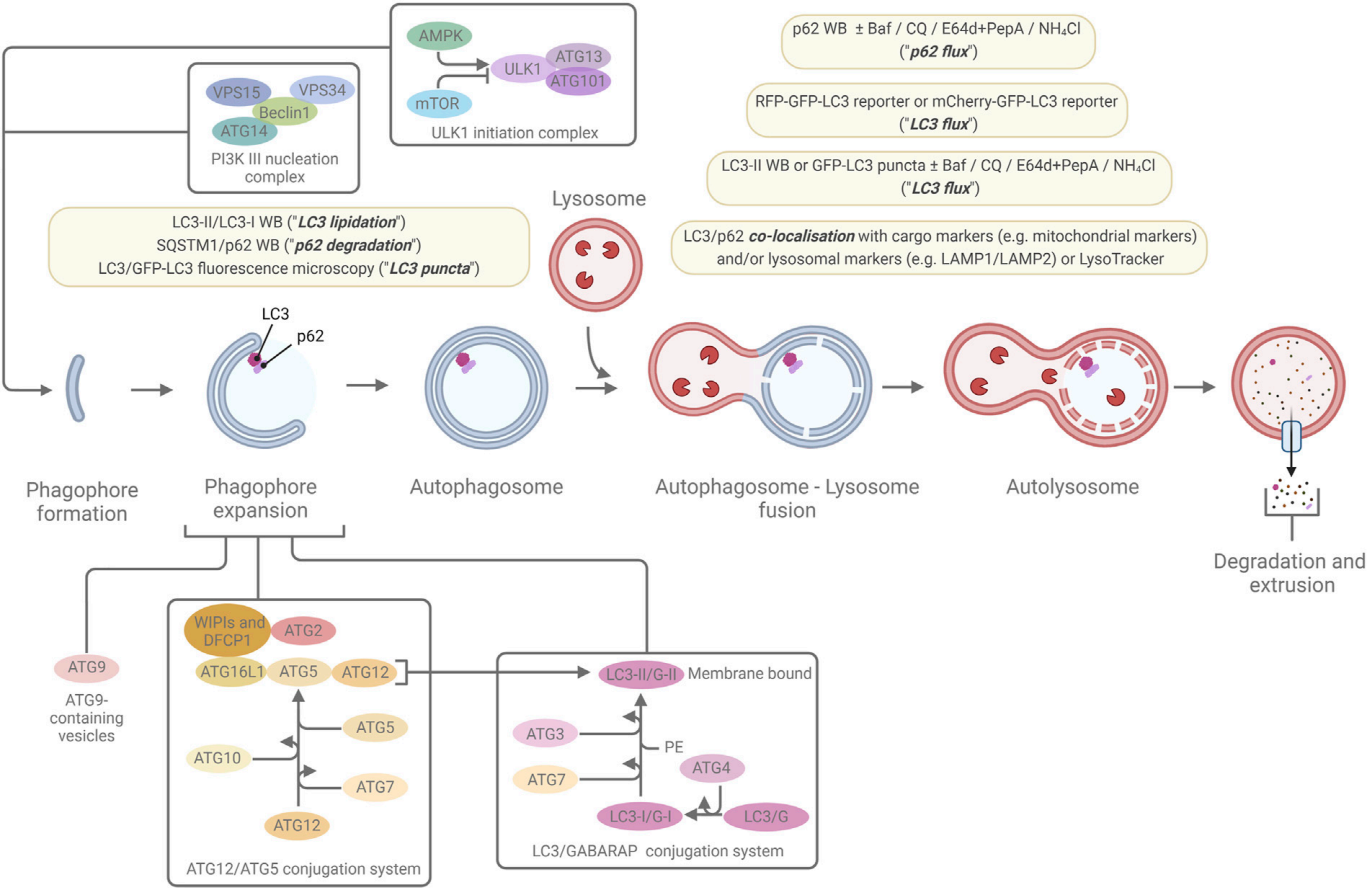
The macroautophagic pathway and the most commonly used marker-based autophagy assays. A simplified overview of the macroautophagic pathway and some of the major proteins, protein complexes, and conjugation systems that are involved in autophagosome formation, are shown. The light-yellow boxes highlight the most common ways to monitor macroautophagy through marker-based assays that indicate levels of LC3 lipidation, p62 degradation, LC3 puncta, LC3 flux, p62 flux, and LC3 or p62 co-localisation with cargo marker or lysosomal markers. For further details, see the main text and references within.

Macroautophagy (from now on referred to simply as “autophagy” unless otherwise specified) is distinct from the other two types of autophagy in that the site of sequestration occurs away from the limiting membrane of the lysosome and, instead, involves the formation of double/multi-membrane cytoplasmic vacuoles which transport cargo to the lysosome. These sequestering vacuoles, termed autophagosomes form *de novo* through expansion instead of budding from a pre-existing organelle containing cargo (Yang and Klionsky, 2009). The initiation step frequently occurs in association with an endoplasmic reticulum subdomain called the omegasome (although there importantly also are other sites and other membrane sources for autophagy initiation), which is enriched for the lipid phosphatidylinositol 3-phosphate (PI(3)P) (Axe et al., 2008). The machinery for autophagosome formation (Figure 2), is highly conserved and contains two major initiation complexes: the ULK1 (unc-51 like autophagy initiating activating kinase 1) complex and the class III PI-3-kinase complex 1 (PI3K3-C1) (Mizushima et al., 2011; Hurley and Schulman, 2014; Suzuki et al., 2017). The membrane then begins to expand and creates the primary double/multi-membrane sequestering structure referred to as the “phagophore” (Seglen, 1987; He and Klionsky, 2009). The expansion of the phagophore is driven by the PI(3)P binding WIPI-1-4 proteins and the ATG (autophagy-related)8 family and ATG12 conjugation systems (altogether involving the following ATG proteins in mammals: ATG3, ATG4A-D, ATG5, ATG7, ATG10, ATG12, ATG16L1, and the mammalian ATG8 (mATG8) homologues MAP1LC3A, MAP1LC3B, MAP1LC3C, GABARAP, GABARAPL1, and GABARAPL2).

MAP1LC3B (microtubule-associated protein 1 light-chain 3B; commonly referred to as LC3B, or simply LC3) and the other mATG8 are cleaved by ATG4 proteases, exposing a C-terminal glycine residue. The cleaved forms (termed mATG8-I, for instance LC3-I or GABARAP-I) can then be conjugated to phosphatidylethanolamine (PE) (Kabeya et al., 2004; Sou et al., 2006) (termed “lipidation”) by sequential ubiquitylation-like reactions catalysed by ATG7 (E1-like enzyme), ATG3 (E2-like enzyme), and the ATG5/ATG12-ATG16L1 complex to generate a membrane-bound form of mATG8-I, referred to as mATG8-II (for instance LC3-II or GABARAP-II) (Kabeya et al., 2000; Tanida et al., 2001; Tanida et al., 2002; Tanida et al., 2004). Initially, both the LC3- and GABARAP subfamilies of the mATG8 proteins were thought to be essential for autophagosome formation (Weidberg et al., 2010), however, recent studies by us and others indicate that the GABARAPs play a dominant role, and that the LC3s are frequently not required for the autophagic pathway to go to completion (Szalai et al., 2015; Nguyen et al., 2016; Vaites et al., 2018; Luhr et al., 2019; Sønstevold et al., 2021). An important other function of the mAGT8s is to recruit various types of autophagy receptors to the inner phagophore membrane via interaction with specific motifs/regions in the receptors, the best described being the so-called LC3/ATG8-interacting region/motif (LIR/AIM). This interaction can mediate selective autophagy when the autophagy receptors in turn are bound to specific cargo. Selective autophagy receptors include: i) soluble receptors that bind to ubiquitinated cargo, e.g., SQSTM1 (commonly referred to as p62), NBR1, NDP52, TAX1BP1, and OPTN, ii) soluble, ubiquitin-independent receptors, e.g., NCOA4 and NUFIP1, and iii) membrane-bound receptors, e.g., the mitochondrially-residing receptors NIX, BNIP3, and FUNDC1, and the ER-residing receptors FAM134B, RTN3L, CCPG1, SEC62, TEX264 and ATL3 (Lamark and Johansen, 2021).

During late-stage expansion of the phagophore, the membrane bends to generate a spherical structure and, upon completion, the phagophore fully surrounds its cargo and forms a closed structure called the “autophagosome.” The outer membrane of the autophagosome may fuse with a lysosomal membrane, the product of which is referred to as an “autolysosome” (Yang and Klionsky, 2009). The inner autophagosome membrane and the autophagic cargo are subsequently degraded due to exposure to the acidic lumen and hydrolases of the lysosome. Component parts are transported back to the cytoplasm via lysosomal permeases and can be utilized by the cell or the organism as building blocks or to generate energy (Yorimitsu and Klionsky, 2005).

As the names imply, the ATG proteins were identified through their roles in autophagy. Importantly, however, it has become increasingly evident that most, if not all, ATG proteins also have non-autophagic functions (Velikkakath et al., 2012; Elgendy et al., 2014; Rohatgi et al., 2015; Kaizuka and Mizushima, 2016; Nunes et al., 2016; Yang et al., 2016; Cadwell and Debnath, 2018; Galluzzi and Green, 2019; Hu et al., 2020; Fang et al., 2021; Mailler et al., 2021; Zhang et al., 2022a; Hamaoui and Subtil, 2022; Tedesco et al., 2024; Tran et al., 2024). This includes the by far most used autophagic marker LC3B (Baisamy et al., 2009; Hanson et al., 2010; Chung et al., 2012; Ramkumar et al., 2017; Lindner et al., 2020; Nieto-Torres et al., 2021; Yoon et al., 2024), which originally was identified as a microtubule-associated protein in the 1980s (Vallee and Davis, 1983) before its revelation as a mammalian homologue of yeast ATG8 in 2000 (Kabeya et al., 2000). Also, autophagy receptors such as p62 have non-autophagic functions (Moscat et al., 2007; Moscat et al., 2016). Together, this has important implications for the interpretation of results that are based on methods which employ ATGs like LC3 or other markers to explore the function of autophagy, as described further below.

## 3 Methods to monitor autophagy

In order to critically review the role of autophagy in prostate cancer formation and therapy, it is essential to be aware of the principles of various methods that are used to monitor autophagy, and the difference between them. To that end, we here provide a brief overview of various autophagy assays, with key references and a particular emphasis on methods that have been employed in publications that specifically have explored the role of autophagy in prostate carcinogenesis and prostate cancer therapeutics.

**FIGURE 3:**
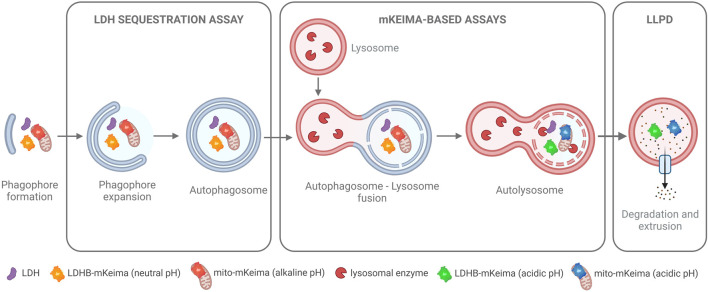
Functional Autophagy Assays. An overview of cargo-based methods to measure autophagy at different steps of the pathway. The LDH sequestration assay measures the sequestration of the ubiquitously expressed, soluble cytosolic protein lactate dehydrogenase (LDH) into autophagosomes. The mKeima-based assays measure the autophagic flux of either cytosol (with the LDHB-mKeima probe) or of specific cargo (e.g., of mitochondria as illustrated here with the mito-mKeima probe). Upon flux of the cargo probes to the acidic environment of the autolysosome, mKeima, which is resistant to lysosomal enzymes, shifts its fluorescence excitation maximum, thus allowing detection and ratiometric quantification of the autophagic flux of the cargo probes. The long-lived protein degradation (LLPD) assay is an endpoint assay that utilizes radiolabeling of cellular proteins to track the extrusion of amino acid products from autolysosomes into the cytoplasm, which occurs as a result of the autophagic degradation of long-lived proteins in the autolysosomes. For further details, see the main text and references within.

In the first era of autophagy research (1950s–1980s), autophagy was predominantly assessed by morphometric analyses of electron microscopy images. To date, this remains one of the gold standard methods. However, since autophagy is a dynamic process, determination of autophagic activity requires quantitative analyses of cargo flux through the pathway. In the late 1970s and 80s, Per O. Seglen identified the first autophagy inhibitors and pioneered the development of functional assays to quantify bulk autophagic flux (Seglen et al., 1979; Seglen and Gordon, 1982; Kopitz et al., 1990; Seglen et al., 2009; Seglen et al., 2015), some of which have been revived, optimized, and extensively validated by us, e.g., the LDH sequestration assay (Luhr et al., 2018a) and the long-lived protein degradation assay (Luhr et al., 2018b) (Figure 3). Later, genetically engineered cargo reporters, such as those that utilize the fluorescent coral protein mKeima, have been developed to monitor both bulk and selective autophagy (Katayama et al., 2011; An and Harper, 2018; Engedal et al., 2022) (Figure 3).

Although cargo-based autophagy methods thus have been re-introduced to autophagy research, the last two decades have been overwhelmingly dominated by the use of autophagic markers to monitor autophagy (Figure 2)—primarily LC3 and secondarily p62. Cellular LC3-II levels can be assessed by Western blotting, since LC3-II travels quicker through SDS-PAGE gel than its cytosolic counterpart (LC3-I). Accumulation of LC3-II on phagophores and autophagosomes can also be detected by fluorescence microscopy in the form of “LC3-positive puncta.” Whilst most phagophore-interacting proteins dissociate during autophagosome formation, LC3s and GABARAPs stay attached to the inner autophagosome membrane throughout the pathway until they are degraded in the autolysosomes. Similarly, p62, which is an autophagy receptor that on the one hand interacts with LC3s and GABARAPs and on the other hand can interact with specific cargo, stays associated with the inner autophagosome membrane until degraded in the autolysosomes. Therefore, by assessing LC3-II or p62 levels or LC3- or p62-positive puncta in the absence or presence of lysosomal inhibitors such as Bafilomycin A1 (Baf A1; blocks the lysosomal proton pump and thereby neutralises lysosomal pH and therefore autolysosomal function), Chloroquine (CQ; weak base that temporarily neutralises lysosomal pH and blocks autophagosome-lysosome fusion (Lu et al., 2017; Mauthe et al., 2018)), or E64d + Pepstatin A (PepA) (lysosomal protease inhibitors), the autophagic flux of LC3 and p62 can be inferred. Such measurements are frequently used as proxies for autophagic cargo flux. However, there are a number of important limitations and potential confounding factors in the use of autophagic markers like LC3 and p62 in assessing autophagic activity (Klionsky et al., 2021) including potential influences from transcriptional and translational regulation of LC3 and p62 (Engedal et al., 2013; Klionsky et al., 2021), conjugation of LC3 to other membrane compartments than phagophores/autophagosomes (Malhotra, 2013; Pimentel-Muiños and Boada-Romero, 2014; Durgan et al., 2021), conjugation of LC3 to proteins (termed “protein ATG8ylation”) (Carosi et al., 2021), puncta formation due to LC3/p62 aggregation in the absence of phagophores (Szeto et al., 2006; Kuma et al., 2007; Runwal et al., 2019), a high degree of uncertainty in the correlation between LC3/p62 marker flux versus actual cargo flux, and that LC3 and p62 have many other cellular functions than autophagy (Moscat et al., 2007; Baisamy et al., 2009; Hanson et al., 2010; Chung et al., 2012; Moscat et al., 2016; Ramkumar et al., 2017; Lindner et al., 2020; Kumar et al., 2021; Nieto-Torres et al., 2021; Yoon et al., 2024) and are not always necessary for autophagy (Sala et al., 2014; Szalai et al., 2015; Xu et al., 2015; Nguyen et al., 2016; Vaites et al., 2018; Luhr et al., 2019; Sønstevold et al., 2021). Therefore, the use of autophagic markers alone is insufficient for drawing solid conclusions about autophagic activity (Klionsky et al., 2021). Indeed, upon direct comparison of cargo-based versus LC3-based assays, we have identified conditions where LC3 flux measurements show a complete lack of positive correlation with actual autophagic cargo flux in mammalian cells (Szalai et al., 2015; Sønstevold et al., 2021).

Alterations in autophagy may also be assessed by dyes that stain autophagic vacuoles, such as the acidotropic dyes monodansylcadaverine (MDC), its derivative Monodansylpentane (also referred to as “AUTOdot”), and acridine orange, or with the amphiphilic dye “CYTO-ID”. In general, these dyes display a substantial lack of specificity towards autophagic vacuoles and are not recommended to be used on their own to monitor changes in autophagy (Klionsky et al., 2021).

Various autophagy modulators (inhibitors and activators) are used as tools in the autophagy field. Those mentioned in this review are listed in Table 1. For further reading, methods to monitor autophagy are excellently presented and referenced in the fourth Edition of the autophagy community’s “Guidelines for the use and interpretation of assays for monitoring autophagy” (Klionsky et al., 2021).

**TABLE 1:** Overview of autophagy-modulating drugs mentioned in this review.

Drug	Autophagy modulation	Mechanism of action
Bafilomycin A1 (Baf A1), Concanamycin A (Con A), Pantoprazole	Inhibition	Inhibit lysosomal and autolysosomal function by blocking the v-ATPase lysosomal proton pump
Chloroquine (CQ), Hydroxychloroquine (HCQ)	Inhibition	Weak base that inhibits autophagosome-lysosome fusion
E64d, Pepstatin A (PepA)	Inhibition	Inhibit lysosomal proteases
3MA	Inhibition	Inhibits autophagy via PI3K class III inhibition
ULK101	Inhibition	Inhibits ULK1 and ULK2
Rapamycin, everolimus	Activation	Activate autophagy via mTOR inhibition
Trehalose	Activation	Activates autophagy via an mTOR-independent mechanism

## 4 The role of autophagy in cancer—a brief overview

Traditionally, autophagy has been viewed as a pathway that enables the survival of cells, in particular under starvation or energy deprivation conditions. This is ascribed to its function in nutrient recycling and metabolic adaptation (Lum et al., 2005; Onodera and Ohsumi, 2005; Lecker et al., 2006; Poillet-Perez et al., 2018). It has been suggested that autophagy plays a tumour suppressive role in non-transformed cells via removing damaged organelles, reactive oxygen species, and misfolded proteins, which otherwise could promote cellular transformation (Gozuacik and Kimchi, 2004; Mizushima, 2007; Ávalos et al., 2014; Das et al., 2020). This is in line with early work from the group of Per O. Seglen, who almost 40 years ago demonstrated that autophagy is downregulated during liver carcinogenesis in rats (Schwarze and Seglen, 1985; Kisen et al., 1993), marking the first documented association between autophagy and cancer. A putative genetic link was identified ∼15 years later by Beth Levine’s lab who showed that monoallelic loss of *Becn1* (homologue of yeast *ATG6*) can contribute to several types of malignancies in mice (Liang et al., 1999; Qu et al., 2003). Another ∼12 years later, the group of Noboru Mizushima observed development of benign liver tumours in mice with either mosaic deletion of *Atg5* or liver-specific deletion of *Atg7* (Takamura et al., 2011). The tumour cells in these mice display swelling of the mitochondria, accumulation of p62, oxidative stress and genomic damage responses (Takamura et al., 2011), indicating the global effect a defect in autophagy can have on cellular transformation. In humans, loss of *BECN1* is limited to a few cancer types, like those of the breast and ovary, and co-occurs with the loss of *BRCA1* (Laddha et al., 2014). Furthermore, at a general level, copy-number alterations or mutations in ATG genes are very rare across human cancers (Lebovitz et al., 2015). Therefore, cancer-related changes in autophagic activity levels predominantly have other underlying causes, which are yet to be defined. Although the above-mentioned and other studies (Galluzzi et al., 2015) support the notion that autophagy suppresses malignant transformation, conclusive encompassing evidence is still lacking, in part due to the increasing realisation of the important non-autophagic functions of the ATGs.

With the progression of cancer, it has been proposed that autophagy proficiency might be restored and elevated in the cancer cells, and/or in other cells in the tumour microenvironment and host tissues, to fulfill the metabolic needs of the tumour cells to survive and grow (Galluzzi et al., 2015; Sousa et al., 2016; Katheder et al., 2017). Additionally, autophagy may be utilized by the cancer cells to survive therapeutic insults (Moosavi and Djavaheri-Mergny, 2019; Chang and Zou, 2020; Mele et al., 2020; Vempati and Malla, 2020; Ahmadi-Dehlaghi et al., 2023). Conversely, it has been reported that autophagy can play tumour suppressive roles also in established tumours. For instance, autophagy may enhance anti-tumour immune responses (Zhong et al., 2016), and limit cancer metastasis (Marsh et al., 2021). Moreover, autophagy may contribute to cell death under various conditions, including in response to some types of anti-cancer therapies (Linder and Kögel, 2019). In sum, the function of autophagy in cancer appears to be highly complex and may have different roles in different cancer types, cancer stages and conditions.

## 5 Autophagy in prostate carcinogenesis

It is unclear whether autophagy levels are altered during prostate tumorigenesis and how that may affect cancer development and progression. However, analyses of autophagic markers and links between expression levels of ATGs and prostate carcinogenesis have been explored in various settings, including patient tissue, mouse models, and cell lines. Moreover, studies have used marker-based assays to investigate how major genetic drivers of prostate carcinogenesis, e.g., androgens and TP53, affect autophagy. These studies are discussed below (in Section 5.1 and 5.2, respectively).

### 5.1 Links between expression levels of ATGs and prostate carcinogenesis

An early study indicated loss of heterozygosity of the *BRCA1* loci and other loci on chromosome 17q in human prostate cancer (Gao et al., 1995), suggesting monoallelic loss of *BECN1*, which is located at 17q23.31 (Liang et al., 1999) (Table 2). However, a more detailed study on a larger set of patients (from the TCGA cohort) revealed that loss of *BECN1* is a very rare event in prostate cancer (Laddha et al., 2014). Moreover, *Becn1* ^+/−^mice do not develop prostate tumours, unlike the spontaneous generation of liver and lung carcinomas observed in these mice (Liang et al., 1999; Qu et al., 2003). On the other hand, in a genetically engineered mouse model of prostate cancer with inducible prostate-specific deficiency in the *Pten* tumour suppressor gene (which is frequently lost in human prostate cancers), combined prostate-specific deficiency in *Atg7* led to a delay in prostate tumour development and growth of both primary and castrate-resistant tumours (Santanam et al., 2016). These results may indicate a tumour promoting role of autophagy in prostate carcinogenesis. However, before concluding this, it is imperative to examine the effects of depletion of several other ATGs, as well as their effect in different models of prostate cancer. It is important to note that ATG7 has several non-autophagic functions (DeSelm et al., 2011; Lee et al., 2012; Chen et al., 2023; Deng et al., 2023), and in relation to this, an alternative explanation for the delayed development and growth of the *Pten* deficiency-induced mouse prostate tumours mentioned above (Santanam et al., 2016), could for instance, be related to the autophagy-independent effect that ATG7 has upon binding to the tumour suppressor TP53, which results in enhanced apoptotic cell death in the absence of *Atg7* (Lee et al., 2012). It should also be noted that prostate cancer has not been associated with alterations in ATG7 expression or function.

**TABLE 2:** Overview of published studies on the role of autophagy in **prostate carcinogenesis**.

Model/Material	Autophagy intervention	Findings	Suggestive role of autophagy	Study
Genetically engineered mouse model with inducible prostate-specific Pten deficiency	*Atg7* deficiency	Pten deficiency resulted in development of prostate tumours in the mice. Combined loss of *Pten* and *Atg7* delayed prostate tumour development and growth	**Tumour promoting** (Unclear which carcinogenesis stage *Atg7* loss affected)	Santanam et al. (2016)
LNCaP cells (*in vitro* and in NOD/SCID mouse xenograft)	sh*ATG5* (1 shRNA, stably transfected)	sh*ATG5* led to increased LNCaP cell migration, invasion and wound healing *in vitro*, and appearance of lung metastases in xenografts *in vivo*	**Tumour suppressive** (Cancer progression)	Shi et al. (2022)
DU145 cells	Overexpression of HA-tagged ATG5	Reconstitution of ATG5 expression in DU145 cells restored LC3 lipidation and LC3 puncta formation, and led to enhanced *in vitro* cell proliferation and migration as assessed by wound healing	**Tumour promoting** (Cancer progression)	Peng et al. (2021)
Prostate tumour tissue from 107 (Kim et al., 2011) and 28 (Kharaziha et al., 2015) PCa patients	-	ATG5 protein was frequently overexpressed in prostate tumours, but did not correlate with pathological parameters such as Gleason grade. Loss of ATG5 observed in 18% of the castration-resistant patients (Kharaziha et al., 2015)	**Tumour suppressive** in a small subset of patients (Cancer progression)	Kim et al. (2011), Kharaziha et al. (2015)
Tumour tissue from radical prostatectomy of patients with clinically localised PCa (160 patients)	-	High protein expression of ULK1 (but not of ATG5, BECLIN1, LC3, or ATG9) associated with biochemical recurrence	**Tumour promoting** (Cancer progression)	Liu et al. (2015)
Tumour tissue from PCa patients with metastatic disease and receiving ADT (198 patients)	-	High concomitant protein expression of ULK1+LRPPR (but not of ATG5, BECLIN1, or LC3B) correlated with serum PSA levels, Gleason score, PSA levels following ADT, and amount of metastatic lesions	**Tumour promoting** (Cancer progression)	Zhang et al. (2017)
LNCaP cells and tumour tissue from radical prostatectomy of PCa patients with no previous treatment (135 patients)	si*FIP200* (1 oligo)	si*FIP200* promoted cell cycle progression and reduced cell death. PCa patients who had undetectable FIP200 protein expression in their tumours showed increased biochemical recurrence	**Tumour suppressive** (Cell cycle, cell death, cancer progression)	Li et al. (2014)
Publicly available data from the MSKCC Prostate Oncogenome Project (140 patients)	-	Low tumour expression of *ATG16L1* mRNA correlated with high Gleason score, metastasis, and poor survival	**Tumour suppressive** (Cancer progression)	Huang et al. (2015)
Publicly available PCa data from the Oncomine website	-	Progressive loss of *GABARAPL1* with increased Gleason scores. Low *GABARAPL1* mRNA expression correlated with poor survival	**Tumour suppressive** (Cancer progression)	Su et al. (2017)
LNCaP and VCaP cells	3MA, Baf A1, CQ, si*ATG7* (3 oligos)	Androgens increased LC3-II levels, GFP-LC3 puncta, and mCherry-GFP-LC3 flux. Autophagy intervention reduced androgen-mediated cell proliferation	**Tumour promoting** (Cell proliferation)	Shi et al. (2013)
LNCaP and VCaP cells. Gene expression data from TCGA and three other publicly available clinical prostate cancer cohorts	si*ATG4B*, si*ATG4D*, si*ULK1*, si*ULK2* (2 oligos) si*TFEB* (1 oligo). ATG4B, ATG4D, ULK1, ULK2, or TFEB overexpression	Androgens increased ATG4B, ATG4D, ULK1, ULK2, and TFEB expression. A combined gene expression score of these 5 genes was elevated in metastatic disease. Knockdown of any of the 5 genes diminished androgen-induced cell proliferation. Overexpression of any of the 5 genes enhanced cell proliferation independently of androgen. High mRNA expression of *ATG4B*, *ATG4D*, *ULK1* or *ULK2* correlated with poor prognosis	**Tumour promoting** (Cell proliferation, cancer progression)	Blessing et al. (2017)
PC3, LNCaP, and CWR22R cells	-	Reconstitution of AR expression in PC3 cells inhibited autophagosome formation (electron microscopy) and decreased MDC staining and GFP-LC3 puncta. Knockdown of *AR* in LNCaP and CWR22R cells increased MDC staining and GFP-LC3 puncta	**Tumour suppressive** (Inverse correlation with cell growth)	Jiang et al. (2012)
LNCaP cells	-	Androgens decreased serum starvation-induced LC3-II flux and mRFP-GFP-LC3 flux via upregulation of Grp78/BiP, which counteracted starvation-induced cell death	**Tumour suppressive** (Correlation with cell death)	Bennett et al. (2010)
Prostate tumour tissue from 274 untreated PCa patients undergoing radical prostatectomy and 36 CRPC patients	CQ, ULK101 (ULK inhibitor)	*TP53* loss-of-function mutations were associated with increased LC3B, ULK1 and BECLIN1 expression in CRPC tumour specimens. Androgen-independent tumour organoids were highly vulnerable to CQ or ULK101 in combination with enzalutamide	**Tumour promoting** (Cancer progression)	Zhang et al. (2022b)

In terms of cell line studies, 3 human prostate cancer cell lines are predominantly utilized in the prostate cancer field, namely, the hormone-responsive LNCaP cells (originating from a lymph node metastasis, but retaining many of the phenotypes of primary disease) and the hormone-insensitive cell lines PC3 and DU145 (originating from bone and brain metastases, respectively). LNCaP cells have less aggressive traits than PC3 and DU145 in terms of poorer *in vitro* proliferation, migration and invasion abilities, as well as lower tumorigenicity and metastatic potential in mouse models (Sobel and Sadar, 2005; Shi et al., 2022). PC3 and DU145 cells are AR-negative (Sobel and Sadar, 2005) and therefore not prototypic CRPC cells, since clinically developed CRPC cells predominantly retain AR expression and activity. CRPC versions of LNCaP cells have been developed from LNCaP mouse xenografts, where resistant xenograft tumour cells that appeared after castration have been termed C4 (Wu et al., 1994). A second round of xenografting, where C4 cells were xenografted in castrated mice produced castration-resistant cells with metastatic potential, termed C4-2. Xenografting of C4-2 cells in castrated mice produced tumour cells that had metastasised to bone and are termed C4-2B (Thalmann et al., 1994). The LNCaP-C4 model resembles the clinical progression from androgen-dependence to castration-resistance, where the CRPC C4 cell lines retain AR expression and activity (Dehm and Tindall, 2006; Decker et al., 2012). More recently, the VCaP cell line was generated from a vertebral metastasis of a patient with CRPC (Korenchuk et al., 2001). VCaP is androgen-responsive and expresses high amounts of AR (Korenchuk et al., 2001; van Bokhoven et al., 2003).

Interestingly, due to alternative *ATG5* mRNA splicing, DU145 cells do not express any functional ATG5 protein, and therefore display autophagy defects as assessed by LC3/p62 Western blotting, LC3 immunostaining, and LDH sequestration (Ouyang et al., 2013; Luhr et al., 2018a; Wible et al., 2019; Peng et al., 2021). It has been proposed that the defective autophagy is a key contributing factor to the highly migratory and metastatic phenotype of DU145 cells, since stable knockdown of *ATG5* in LNCaP cells, which reduced LC3-II levels and elevated p62 protein levels, led to increased LNCaP cell migration, invasion and wound healing *in vitro*, and a marked appearance of LNCaP lung metastases in an *in vivo* NOD/SCID mouse model (Shi et al., 2022). This indicates that loss of autophagy in prostate cancer cells promotes the metastatic process and thus prostate cancer progression. However, since ATG5 has several highly cancer-relevant non-autophagic functions (Yousefi et al., 2006; Maskey et al., 2013; Guo et al., 2017; Li et al., 2021), it will be essential to evaluate whether the increase in migration, invasion and metastasis that was observed upon *ATG5* knockdown also occurs upon knockdown of other central ATGs. Moreover, the proposal above is apparently in conflict with the results from another study, which reported that overexpression of wild type HA-tagged ATG5 in DU145 cells, which restored LC3 lipidation, LC3-II flux (as assessed by LC3 WB ± CQ) and LC3 puncta formation, led to enhanced *in vitro* cell proliferation and migration as assessed by wound healing (Peng et al., 2021). It remains to be determined how overexpression of HA-ATG5 affects the non-autophagic functions of ATG5, as well as the effect the overexpression will have on other assays for migration and invasion *in vitro*, and on metastasis in *in vivo* models. It also remains to be determined whether different degrees of ATG5 overexpression would have different cellular effects. Of relevance, two reports have indicated that ATG5 protein is frequently highly overexpressed in prostate tumours compared to normal prostate tissue (Kim et al., 2011; Kharaziha et al., 2015). The overexpression did not correlate with pathologic parameters such as Gleason grade, and no mutations in *ATG5* were found (Kim et al., 2011). Of note, however, loss of ATG5 was observed in 18% of the castration-resistant patients in one of the studies (Kharaziha et al., 2015). Although a rather limited number of patient samples were included in the latter report (only 28 samples were quantified), overall, the results warrant additional studies on the deregulation of ATG5 protein expression in prostate cancer. At the mRNA level, TCGA data reveal that the average expression of full-length *ATG5* mRNA is increased in prostate tumours compared to normal prostatic tissue (Wible et al., 2019). However, also at the mRNA level, a small subset of patients show reduced or complete loss of *ATG5* (Wible et al., 2019).

As opposed to that observed for ATG5, high tumour protein expression of ULK1 has been found to significantly associate with biochemical recurrence following radical prostatectomy of prostate cancer patients (Liu et al., 2015). Similarly, another study found that high concomitant expression of ULK1 and the mitochondrion-associated protein LRPPR correlated with serum prostate-specific antigen (PSA) levels, Gleason score, PSA levels following ADT, and the amount of metastatic lesions (Zhang et al., 2017). However, it is unclear whether these associations are related to alterations in autophagy levels or not, since changes in the expression of other ATG proteins (ATG5, BECLIN1, LC3B, or ATG9) did not show any significant correlations with any clinicopathological parameters (Liu et al., 2015; Zhang et al., 2017). Moreover, ULK1 exerts a number of non-autophagic functions (Joo et al., 2016; Joshi et al., 2016; Wang and Kundu, 2017; Saleiro et al., 2018; Wu et al., 2020; Rajak et al., 2022; Liang et al., 2023), which may be altered upon its increased expression, and overexpression of ULK1 may, somewhat counterintuitively, actually result in reduced autophagy levels (Chan et al., 2007). Correlations between ULK1 tumour expression levels and clinical parameters are therefore very difficult to interpret.

A potential role of *decreased* autophagy in promoting prostate cancer progression has been suggested through correlations between low prostate tumour expression of the core ATG genes *FIP200*, *ATG16L1*, or *GABARAPL1* and poor prognosis in clinical prostate cancer cohorts (Li et al., 2014; Huang et al., 2015; Su et al., 2017). It is important to note also here that it remains to be determined whether, and to what extent, the decreased expressions observed translate into alterations in functional autophagy. Of note, however, we have previously found that knockdown of *FIP200* strongly reduces functional autophagy in LNCaP cells, as assessed by the LDH sequestration and long-lived protein degradation assays (Seglen et al., 2015; Luhr et al., 2018a). Furthermore, *FIP200* silencing with an siRNA oligo was reported to promote cell cycle progression and reduce cell death in LNCaP, suggesting a tumour suppressive function of autophagy in these cells, in concordance with the observed increased biochemical recurrence in prostate cancer patients who had undetectable FIP200 protein expression in their tumours (Li et al., 2014). It is however very important to note that FIP200 has various non-autophagic functions that are highly relevant to both cell proliferation and cell death (Gan and Guan, 2008; Chen et al., 2016).

### 5.2 Links between genetic drivers of prostate carcinogenesis and autophagy

Androgens, which play a crucial role in prostate carcinogenesis, have been shown to increase LC3-II levels and GFP-LC3 puncta, as well as mCherry-GFP-LC3 flux in LNCaP and VCaP cells (Shi et al., 2013). Moreover, androgen-stimulated cell proliferation was sensitive to the autophagy-inhibiting drugs Baf A1, 3-Methyladenine (3MA) and CQ, or siRNA-mediated knockdown of *ATG7* (Shi et al., 2013). 3MA is a pan-PI3K inhibitor that blocks autophagy through PI3K class III inhibition (Seglen and Gordon, 1982; Blommaart et al., 1997). In a follow-up study (Blessing et al., 2017), androgens were found to increase the expression of ATG4B, ATG4D, ULK1 and ULK2, as well as the transcription factor TFEB, which regulates genes involved in autophagy and lysosomal biogenesis. In clinical prostate cancer cohorts, a combined gene expression score of these 5 genes was increased in metastatic disease. Moreover, prostate adenocarcinoma patients from the TCGA dataset displaying a tumour mRNA expression level greater than 2-fold above the mean of either *ATG4B*, *ATG4D*, *ULK1,* or *ULK2*, had poor prognosis. Finally, knockdown of *TFEB* or any of the 4 *ATG* genes diminished androgen-mediated proliferation of prostate cancer cell lines, and overexpression of any of the 5 genes increased cell proliferation independently of androgens (Blessing et al., 2017). These findings suggest that androgens promote prostate cancer growth and progression via an AR-mediated increase in autophagy. However, it remains to be determined whether alterations in the expression of any of the 5 genes identified lead to changes in functional autophagic activity. For instance, there is a high degree of redundancy among *ATGA*, *ATG4B*, *ATG4C* and *ATG4D* (Agrotis et al., 2019), and overall autophagic flux appears to be unchanged in *Atg4D* knockout mice (Tamargo-Gómez et al., 2021). Furthermore, ULK2 is not always necessary for autophagy (due to redundancy with ULK1), and, as mentioned above, overexpression of ULK1 can lead to inhibition of autophagic activity (Chan et al., 2007). Adding to the complexity, other studies performed in prostate cancer cell lines reported that AR efficiently blocks mTOR-inhibitor-induced generation of autophagosomes (Jiang et al., 2012), and in another study, androgens were found to decrease serum starvation-induced LC3-II flux and mRFP-GFP-LC3 flux via upregulation of the endoplasmic reticulum chaperone glucose-regulated protein 78/BiP, with the effect of AR being linked to counteraction of starvation-induced cell death (Bennett et al., 2010). This suggests an autophagy-inhibitory role of AR, which might promote prostate cancer progression by preventing excessive, cell death-promoting autophagy during nutrient limitation in the tumour microenvironment (Eisenberg-Lerner et al., 2009; Bennett et al., 2010).

Loss of *TP53* or acquisition of *TP53* mutations has previously been implicated in driving metastatic castration-resistant prostate cancer (Gundem et al., 2015; Hong et al., 2015). Further research has indicated an association between *TP53* loss-of-function mutations and increased levels of LC3B, ULK1 and BECLIN1 in castration-resistant prostate tumour specimens, and androgen-independent tumour organoids were found to be highly vulnerable to the autophagy inhibitors CQ or ULK101 (an ULK inhibitor) in combination with the anti-androgen enzalutamide (Zhang et al., 2022b). It remains to be determined whether and how the changes in LC3B, ULK1 and BECLIN1 expression translates into alterations in autophagic activity or capacity. Moreover, as a general cautionary note, none of the existing autophagy-inhibitory drugs are specific, and thus the observed reduction in organoid growth could alternatively arise from non-autophagy related effects. Additional tests involving genetic interference with different ATGs would help clarify the role of autophagy in this setting.

In summary, there are several indications that alterations in autophagy may strongly affect the development and progression of prostate cancer, but more research is needed to elucidate its specific effects, as well as to whether, and in which contexts, autophagy plays a tumour suppressor or pro-oncogenic role during prostate carcinogenesis.

## 6 Autophagy in prostate cancer therapeutics

Several studies have explored the role of autophagy in prostate cancer therapy, particularly in the context of the two main non-surgical treatments for advanced prostate cancer; ADT and radiation, but also with regards to chemo- and targeted therapeutics. For an overview, see Table 3 and the remainder of this chapter.

**TABLE 3:** Overview of published studies on the role of autophagy in **prostate cancer therapeutics**.

Treatment		Cell line and Autophagy intervention	Findings	Suggestive role of autophagy	Study
**ADT** (Androgen-Deprivation Therapy)	**Androgen deprivation** or **Bicalutamide**	LNCaP CQ, Con A, si*ATG5*, si*BECLIN1* (1 oligo pool per target)	ADT increased GFP-LC3 puncta and CYTO-ID staining, and decreased p62 levels. Electron microscopy indicated presence of autophagic vacuoles. LC3 WB ± CQ indicated that ADT did not increase LC3 flux. si*ATG5* or si*BECLIN1* enhanced bicalutamide-induced cell death. CQ or Con A increased androgen deprivation- and bicalutamide-induced cell death	**Cytoprotective**	Boutin et al. (2013)
	**Apalutamide**	LNCaP 3MA, CQ, si*ATG5* (1 oligo)	Apalutamide increased LC3 puncta and AUTOdot staining. LC3-II levels seemed to decrease upon co-treatment with apalutamide + CQ compared to CQ alone. si*ATG5* or treatment with 3MA or CQ enhanced apalutamide-induced cell death	**Cytoprotective**	Eberli et al. (2020)
	**Abiraterone**	LNCaP 3MA, CQ or si*ATG5* (1 oligo)	Abiraterone increased LC3 puncta and AUTOdot staining. LC3 staining and LC3-II levels did not seem to be increased by the combination of abiraterone + CQ compared to CQ alone. si*ATG5* or treatment with 3MA or CQ enhanced abiraterone-induced cell death	**Cytoprotective**	Mortezavi et al. (2019)
		LNCaP, PC3 3MA	Abiraterone strongly decreased the levels of both LC3-I and LC3-II, and strongly reduced the presence of autophagic structures as assessed by electron microscopy	**Undetermined** (Control with 3MA alone lacking)	Ma et al. (2019)
**Radiation**	3–6 Gy	PC3, DU145 Everolimus, si*ATG5* + si*BECLIN1* (1 oligo per target)	Everolimus enhanced the detrimental effect of radiation on colony formation. Simultaneous transfection with si*ATG5* + si*BECLIN1* diminished the radiation-effect on colony formation. Radiation and everolimus increased GFP-LC3 puncta	**Pro-cytostatic** (Radio-sensitising)	Cao et al. (2006)
	0–16 Gy	RM1 Rapamycin, CQ	Rapamycin increased, whilst CQ diminished radiation-induced apoptosis. LC3 WB of cells exposed to radiation ± CQ were inconclusive as to whether radiation increased LC3 flux or not	**Pro-apoptotic** (Radio-sensitising)	Wang et al. (2022)
	8 Gy for PC3 cells, 4 Gy for DU145 cells	PC3, DU145 si*LC3A*, si*LAMP2A* (1 oligo per target)	*LC3A*- or *LAMP2A*-targeting siRNA aggravated the detrimental effects of radiation on cell viability	**Cytoprotective** (Radio-protective)	Koukourakis et al. (2015)
	4 Gy	LNCaP, DU145 si*ATG5* (2 oligos), CQ, glutamine starvation	During glutamine starvation, si*ATG5* or CQ sensitised LNCaP but not DU145 cells to the detrimental effect of radiation on colony formation. LC3-II levels were increased upon glutamine starvation in the presence of CQ, but LC3-positive puncta were not	**Cytoprotective** (Radio-protective)	Mukha et al. (2021)
**Chemo-therapy and targeted therapies**	**Docetaxel**	LNCaP, PC3 3MA	3MA partially protected PC3 cells from docetaxel-induced cytotoxicity, but not LNCaP cells where 3MA was cytotoxic alone	**Pro-cytotoxic**	Pickard et al. (2015)
		C4-2 si*ATG5* (1 oligo)	Docetaxel increased LC3-II levels and number of yellow RFP-GFP-LC3 puncta. si*ATG5* reduced the number of yellow puncta and decreased docetaxel-induced apoptosis	**Pro-cytotoxic**	Zeng et al. (2018)
		DU145 Overexpression of HA-tagged ATG5	Overexpression of HA-ATG5 led to increased apoptosis and decreased cell numbers in docetaxel-treated cells	**Pro-cytotoxic**	Peng et al. (2021)
		PC3, DU145 si*BECLIN1* (1 oligo), 3 MA	3MA or si*BECLIN1* decreased LC3-II/LC3-I ratio levels in PC3 cells, and aggravated the detrimental effect of docetaxel on PC3 and DU145 cell viability. Docetaxel enhanced GFP-LC3 puncta in both cell lines	**Cytoprotective**	Hu et al. (2018)
		LNCaP, PC3, DU145 si*ATG5* (1 oligo), Overexpression of HA-tagged ATG5, 3MA, Trehalose, rapamycin	Cytotoxic effect of docetaxel unaltered by 3MA or si*ATG5* in PC3 cells, or by ATG5-HA overexpression in DU145 cells. Trehalose partially protected, whilst rapamycin aggravated, docetaxel-induced cytotoxicity in PC3 and LNCaP cells. The effects of trehalose and rapamycin in PC3 cells were abolished upon co-treatment with 3MA or si*ATG5*. Trehalose, but not rapamycin led to colocalization of mitochondria with LC3, p62 and lysosomes	**Pro-cytotoxic** (Rapamycin-induced bulk autophagy) **Cytoprotective** (Trehalose-induced mitophagy)	Cristofani et al. (2018)
		PC3 and VCaP (parental and docetaxel-resistant lines) si*BECLIN1*, si*ATG7* (1 oligo per target), *CQ*	Resistant lines expressed less p62 and more LC3-I and -II, correlating with increased FOXM1 levels. CQ enhanced docetaxel-induced apoptosis in the resistant cells only, and not in the parental lines. si*BECLIN1* or si*ATG7* aggravated docetaxel-induced apoptosis in PC3 and VCaP overexpressing FOXM1	**Cytoprotective**	Lin et al. (2020)
	**Olaparib**	LNCaP, C4-2B, PC3 *ATG16L1* CRISPR/Cas knockout	*ATG16L1* CRISPR/Cas knockout aggravated the detrimental effect of olaparib on colony formation. Pre-treatment with rapamycin enhanced LC3 flux and counteracted the negative effects of olaparib on cell proliferation and homologues recombination DNA repair activity in an ATG16L1-dependent manner, and in part by decreasing nuclear p62 levels	**Cytoprotective**	Cahuzac et al. (2022a)
		LNCaP, C4-2B, DU145 (parental and olaparib-resistant lines)	Autophagy pathway genes enriched in olaparib-resistant cell lines (microarray analyses). Higher basal RFP-GFP-LC3 flux in resistant LNCaP and C4-2B cells	**Cytoprotective** (correlation with resistance)	Cahuzac et al. (2022b)
		LNCaP, C4 Rapamycin, CQ	Loss of *ARH3* or *PARP1* associated with olaparib resistance (genome-wide CRISPR-Cas9 knockout screen). Rapamycin aggravated, whilst CQ counteracted, the detrimental effects of olaparib on C4 cell viability in *ARH3* or *PARP1* knockout cells	**Pro-cytostatic**	Ipsen et al. (2022)
	**AZD5363**	PC3, DU145 Baf A1, CQ, 3MA, si*ATG3* or si*ATG7* (1 oligo per target)	AZD5363 inhibited AKT and mTOR activity, and increased LC3-II flux in PC3 cells. Baf A1, CQ, 3MA, si*ATG3* or si*ATG7* increased AZD5363-induced apoptosis in PC3 cells. AZD5363 + CQ co-treatment reduced PC3 and DU145 xenograft tumour growth	**Cytoprotective**	Lamoureux et al. (2013)

### 6.1 Androgen deprivation therapy (ADT)

In the androgen-responsive LNCaP prostate cancer cell line, it was found that androgen deprivation or treatment with the anti-androgen bicalutamide (an AR antagonist) led to accumulation of GFP-LC3 puncta and the presence of double- and multi-membrane structures, as assessed by electron microscopy (Boutin et al., 2013). Moreover, LC3-II/LC3-I ratio levels were increased by androgen deprivation or bicalutamide treatment in the presence of the lysosomal protease inhibitors E64d and PepA. In further support of autophagy promotion, androgen deprivation or bicalutamide treatment decreased p62 levels in an E64d/PepA-sensitive manner, and enhanced CYTO-ID fluorescent staining. In contrast, bicalutamide failed to increase LC3-II levels or the LC3-II/LC3-I ratio in the presence of CQ (Boutin et al., 2013), indicating that bicalutamide does not increase LC3 flux. It should be noted that in these experiments, the treatment with E64d/PepA or CQ lasted for 48 h which can lead to many indirect and non-specific effects in the cells and thereby affect the results (Klionsky et al., 2021). Moreover, no functional autophagy assays were included, and it is uncertain whether the cyto-ID assay specifically and reliably reflects autophagic activity. Therefore, additional experiments are required to draw more firm conclusions on the effect of androgen deprivation or bicalutamide on autophagic activity in LNCaP cells. A protective role of autophagy in bicalutamide-treated LNCaP cells was suggested by the observation that siRNA-mediated knockdown of *ATG5* or *BECLIN1*, which decreased the number of GFP-LC3 puncta and the LC3-II/LC3-I ratio, led to enhanced bicalutamide-induced cell death (Boutin et al., 2013). Moreover, CQ or Concanamycin A (Con A; acts like Baf A1 to block the lysosomal proton pump and thereby neutralise lysosomal pH) increased cell death in both androgen-deprived and bicalutamide-treated cells (Boutin et al., 2013). Thus, autophagy may serve to prevent or limit ADT-induced cell death in LNCaP cells. As a cautionary note, it should be mentioned that CQ and Con A may promote cell death independently of autophagy inhibition. Moreover, siRNAs may promote cell death via off-target effects, and therefore results from using only one siRNA (or only one pool of siRNAs) per target are uncertain without being accompanied by rescue experiments.

Similarly, it has been reported that treatment with 3MA or CQ, or transfection with an *ATG5*-targeting siRNA led to enhanced cell death in LNCaP cells exposed to the AR-antagonistic anti-androgen apalutamide (Eberli et al., 2020) or the androgen biosynthesis inhibitor abiraterone (Mortezavi et al., 2019). While the conclusion about the potential protective role of autophagy in these conditions are associated with the same uncertainties as mentioned above, the effects of apalutamide or abiraterone on autophagic activity in LNCaP cells also remain unclear thus far, since functional autophagy assays have not been used, and inconsistent and seemingly contradictory results have been obtained with non-functional assays. Thus, while LC3 puncta and AUTOdot staining was increased upon treatment with apalutamide or abiraterone in LNCaP cells (Mortezavi et al., 2019; Eberli et al., 2020), LC3-II levels seemed to be decreased upon co-treatment of apalutamide or abiraterone with CQ compared to that observed with CQ alone (Mortezavi et al., 2019; Eberli et al., 2020), and immunofluorescent LC3 staining did not seem to be increased by the combination of abiraterone + CQ compared to CQ alone (Mortezavi et al., 2019). Monitoring autophagy with LC3 or other markers is associated with variation and many potentially confounding effects (Klionsky et al., 2021). Moreover, the AUTOdot dye (Monodansylpentane), which stains lipid droplets (Chen et al., 2017), lacks specificity to autophagy. Further illustrating the apparently conflicting results that can be obtained when using non-functional autophagy assays, another study reported that treatment of LNCaP or PC3 cells with abiraterone strongly decreased the levels of both LC3-I and LC3-II, and nearly abolished the presence of autophagic structures as assessed by electron microscopy (Ma et al., 2019). Whilst these findings contradict those described above on the effect of abiraterone in LNCaP cells (Mortezavi et al., 2019), and are opposite of the ADT-effects observed with androgen deprivation or bicalutamide in LNCaP cells (Boutin et al., 2013), it remains to be determined whether the changes observed with non-functional assays are a result of increased or decreased autophagic cargo flux. In summary, the effect of ADT on autophagy in prostate cancer cells remains to be fully characterised, and whilst there are indications that autophagy may protect against ADT-induced cell death, such studies should be followed up by knockdown (or knockout) of many different ATGs and with more than one siRNA (or guide RNA) per target, to control for the effect of abolishing non-autophagic functions of ATGs and for off-target effects.

### 6.2 Radiation therapy

The role of autophagy in radiation treatment of prostate cancer cells has also been investigated. One of the main actions of therapeutic ionizing radiation is to damage genomic DNA, leading to defective cancer cell proliferation and potentially cell death. In regards to DNA damage, autophagy is able to either directly or indirectly regulate genomic stability through modulation of reactive oxygen species (Karantza-Wadsworth et al., 2007; Mathew et al., 2007; Hewitt and Korolchuk, 2017), through directly targeting double stranded break repair-associated proteins (Hewitt et al., 2016; Wang et al., 2016; Sharma et al., 2018) or through the selective degradation of nuclear components (Park et al., 2009; Dou et al., 2015). However, other mechanisms related to autophagy may lead to increased sensitivity to radiation (Roy et al., 2022). In line with the latter, the rapamycin derivative everolimus, which is expected to activate autophagy via direct mTOR inhibition, enhanced the effect of radiation in reducing the ability of PC3 and DU145 prostate cancer cells to form colonies from single cells (Cao et al., 2006). Furthermore, simultaneous knockdown of *ATG5* and *BECLIN1* rendered radiation less efficient in reducing colony formation (Cao et al., 2006). Although everolimus and radiation increased the number of GFP-LC3 puncta, no LC3 flux measurements or functional autophagy assays were included, and thus it remains to be assessed whether autophagic activity is increased by the treatments in these cells. Furthermore, it was not tested whether the knockdown of *ATG5* and *BECLIN1* was successful in reducing autophagic activity. In relation to findings in the DU145 cell line, it is important to keep in mind that these cells are defective in ATG5-dependent autophagy, as they do not express any functional ATG5 protein due to alternative *ATG5* mRNA splicing (Ouyang et al., 2013; Wible et al., 2019; Peng et al., 2021). Hence, although the radiosensitising and radioprotective effects of everolimus and *ATG5*+*BECLIN1* knockdown, respectively, were more pronounced in PC3 cells than in DU145 cells (Cao et al., 2006), at least part of the effects is likely to be unrelated to changes in autophagy. In support of the notion that autophagy may contribute to the radiosensitivity of prostate cancer cells, rapamycin was found to elevate radiation-induced apoptosis in RM1 murine prostate cancer cells, whilst CQ rendered the cells less sensitive to radiation (Wang et al., 2022). These effects were associated with downregulated expression of the deacetylase SIRT1 upon radiation, which was aggravated by rapamycin and diminished by CQ (Wang et al., 2022). LC3 Western blotting of RM1 cells treated with radiation ± CQ were inconclusive in determining whether radiation increased LC3 flux or not (Wang et al., 2022). In contrast to the two studies mentioned above, another report proposed that autophagy contributes to radioresistance in prostate cancer cells, since siRNA-mediated knockdown of *LC3A* or *LAMP2A* aggravated the detrimental effects of radiation on cell viability in PC3 and DU145 cells (Koukourakis et al., 2015). It is difficult to ascertain whether radiation altered autophagic activity or not in this study, since changes in autophagy were assessed by LC3A/LAMP2A localization and steady-state measurements of LC3A-II, LAMP2A and p62 levels, without the use of any flux measurements or functional autophagy assays. It is also unclear whether *LC3A* or *LAMP2A* knockdown inhibited autophagy, as the effects of the *LC3A*- and *LAMP2A*-targeting siRNAs on autophagic activity were not tested. Furthermore, the ability of the two siRNAs to also radiosensitise DU145 cells, which lack functional ATG5, suggest that their effects may be unrelated to alterations in autophagy. However, another study found that during glutamine starvation, two different *ATG5*-targeting siRNAs sensitised LNCaP but not DU145 cells to the effects of radiation on colony formation (Mukha et al., 2021). The authors proposed that glutamine starvation activates radioprotective autophagy. However, LC3-based assays were inconclusive in determining whether glutamine starvation induced LC3 flux in LNCaP cells, since LC3-II levels were increased upon glutamine starvation in the presence of CQ, but LC3-positive puncta were not. Functional autophagy assays were not used. Moreover, the effect of radiation on autophagy and the result of knocking down other *ATG*s than *ATG5* on radiosensitivity were not assessed. The influence of *ATG5* knockdown on radiosensitivity in the presence of glutamine was also not tested. Taken together, the published reports on the role of autophagy in prostate cancer radiotherapy are incomplete and partly contradictory. More research is required to clarify i) how radiation affects autophagic activity and ii) whether autophagy promotes or counteracts the detrimental effects of radiation in prostate cancer cells.

### 6.3 Chemotherapy and targeted therapeutics

#### 6.3.1 Chemotherapy: Docetaxel

Docetaxel is a chemotherapeutic drug in the class of taxanes, which inhibits microtubule depolymerization and thereby blocks cancer cell division and promotes cell death. Docetaxel is the mainstay first-line treatment of metastatic, castration-resistant prostate cancer. However, limited therapeutic efficacy and development of treatment resistance seriously restrict and shorten the clinical benefits. Several studies have examined how autophagy may influence the effects of docetaxel on prostate cancer cells. One study observed that 3MA partially protected PC3 cells from docetaxel-induced cytotoxicity, but not LNCaP cells, where 3MA was substantially cytotoxic by itself (Pickard et al., 2015). This suggested a pro-cytotoxic role of autophagy in docetaxel-treated cells. In line, another study found that docetaxel-induced cytotoxicity, caspase activity and apoptosis in C4-2 cells was dampened upon transfection with an *ATG5*-targeting siRNA (Zeng et al., 2018). Docetaxel increased LC3-II levels and the number of yellow puncta in C4-2 cells expressing RFP-GFP-LC3. Whilst si*ATG5* strongly reduced the number of yellow puncta, the effect of docetaxel on LC3 flux was not assessed. A pro-cytotoxic role of autophagy in docetaxel-treated cells was likewise indicated in third study, which observed that docetaxel-induced apoptosis and decrease in cell numbers were strongly increased upon overexpression of HA-tagged ATG5 in DU145 cells (Peng et al., 2021). The authors verified that HA-ATG5 overexpression restored LC3 lipidation and flux in the DU145 cells. In contrast, a fourth study reported that treatment of PC3 cells with 3MA or a *BECLIN1*-targeting siRNA, which decreased LC3-II/LC3-I ratio levels, aggravated the detrimental effect of docetaxel on cell viability (Hu et al., 2018). However, very similar results were obtained in the *ATG5*-deficient DU145 cell line, which suggests that the observed effects of 3MA and the *BECLIN1*-targeting siRNA on docetaxel-induced cytotoxicity may have been unrelated to changes in autophagy. Of note, docetaxel was reported to strongly enhance GFP-LC3 puncta in both cell lines, indicating that this was an ATG5-independent phenomenon. In PC3 cells, docetaxel slightly increased LC3-II levels in the presence of CQ, indicating a modest induction of LC3 flux (Hu et al., 2018).

Inconsistent with the four papers described above, a fifth study observed that the detrimental effect of docetaxel on cell viability in PC3 cells was unaltered by treatment with 3MA or transfection with an *ATG5*-targeting siRNA (Cristofani et al., 2018). Moreover, docetaxel-induced cytotoxicity was unchanged upon overexpression of HA-tagged ATG5 in DU145 cells. Docetaxel increased the mRNA and protein expression levels of both LC3 and p62, but no flux measurements were performed. In either case, the putative ability of docetaxel to activate or inhibit autophagy was apparently not sufficient to alter the cytotoxic effects of docetaxel. However, the disaccharide trehalose, which has been proposed to induce autophagy independently of mTOR inhibition (Sarkar et al., 2007), was found to partially protect PC3 and LNCaP cells, but not DU145, from docetaxel. In contrast, the mTOR inhibitor rapamycin aggravated the detrimental effects of docetaxel in PC3 and LNCaP cells, but not in DU145. The effects of trehalose and rapamycin in PC3 cells were abolished upon co-treatment with 3MA or transfection with an *ATG5*-targeting siRNA. Treatment with trehalose, but not rapamycin, led to colocalization of mitochondria (MitoTracker) with LC3, p62 and lysosomes (LysoTracker). This led the authors to propose that trehalose induced its cytoprotective effect via autophagic degradation of mitochondria (mitophagy), whereas rapamycin promoted cell death via bulk autophagy. It should be noted that the authors only used one *ATG5*-targeting siRNA oligo, and that they did not test whether the si*ATG5* reduced trehalose-induced mitochondrial colocalization with LC3, p62 or lysosomes. Moreover, the authors did not examine whether treatment with trehalose led to completion of the mitophagic process, i.e., flux of mitochondria to acidic autolysosomes and their degradation - as opposed to a putative trehalose-mediated block in the fusion of autophagosomes with lysosomes. Of relevance, the autophagy-inducing effect of trehalose has been questioned by data that indicates lack of autophagy induction and instead a trehalose-induced impairment of lysosomal membrane integrity, leading to inhibition of autophagosome–lysosome fusion (Yoon et al., 2017). Finally, a sixth study examined autophagy in partially docetaxel-resistant PC3 and VCaP prostate cancer cells (Lin et al., 2020). Compared to the parental cell lines, the more resistant cells displayed lower expression of p62, and higher expression of LC3-I and -II, along with indications of higher LC3 flux as assessed by Western blotting of CQ-treated cells. Additionally, the resistant lines expressed higher levels of the transcription factor Forkhead box protein M1 (FOXM1), which contributed to resistance as well as to the changes in p62 and LC3 levels. Treatment with CQ led to an increase in docetaxel-induced apoptosis in the resistant, but not the parental cell lines. Moreover, in PC3 and VCaP cells with enforced FOXM1 overexpression, a *BECLIN1*- or an *ATG7*-targeting siRNA aggravated docetaxel-induced apoptosis. Together, this study suggests autophagy as a cytoprotective mechanism that contributes to docetaxel resistance. However, since the results from the six studies mentioned here vary considerably and partly contradict one another, it is impossible to draw a consensus conclusion on the role of autophagy in the regulation of docetaxel cytotoxicity and treatment resistance in prostate cancer cells. In order to obtain more decisive evidence, the use of functional autophagy assays, more than one siRNA per target (and/or rescue experiments) and targeting of several different ATGs along with verification of successful interference with functional autophagy, is needed. Very little is known about the role of autophagy during treatment with chemotherapies other than docetaxel in prostate cancer.

#### 6.3.2 Targeted therapy

Clinically approved targeted therapies for prostate cancer comprise various PARP inhibitors (olaparib, niraparib, rucaparib, and talazoparib) and the PSMA-targeted radioligand therapy lutetium (^177^Lu) vipivotide tetraxetan (Lu-PSMA). There is very limited knowledge about how such therapies affect autophagy in prostate cancer cells and how autophagy may influence their therapeutic efficacy and/or the development of treatment resistance. The exception is for olaparib, where a recent study demonstrated that *ATG16L1* CRISPR/Cas knockout sensitised LNCaP, C4-2B and PC3 cells to the detrimental effect of olaparib on colony formation from single cells (Cahuzac et al., 2022a). Furthermore, pre-treatment with rapamycin, which enhanced LC3 flux, counteracted the negative effects of olaparib on cell proliferation and homologues recombination (HR) DNA repair activity in an *ATG16L1*-dependent manner. This effect of rapamycin was lost in the *ATG16L1* KO cells and rescued upon re-introduction of HA-tagged *ATG16L1*, demonstrating specific relation to *ATG16L1* and not to any off-target effect introduced by the knockout. Mechanistically, the effects of rapamycin and *ATG16L1* KO were linked to variations in nuclear p62 levels, which displayed an inverse correlation with nuclear levels of the FLNA protein that promotes the recruitment of BRCA1 and Rad51 to DNA break sites. Together, this indicates that autophagy plays a protective role by increasing HR-mediated DNA repair in olaparib-treated cells, which may contribute to olaparib resistance. As a cautionary note, however, the direct link to autophagy was not tested with other autophagy inducers than rapamycin or with knockdown/knockout of other ATG genes than *ATG16L1*. Moreover, the effect of olaparib on autophagy was not assessed, and since no functional autophagy assays were employed, it remains to be determined whether a fully functional autophagy pathway would be required for the observed effects. In another study from the same group, microarray analyses revealed that autophagy pathway genes were enriched in olaparib-resistant versions of LNCaP, C4-2B and DU145 cells (Cahuzac et al., 2022b). Moreover, resistant LNCaP and C4-2B cells displayed higher basal LC3 flux as assessed upon transient transfection with an RFP-GFP-LC3 reporter. Contrarily, a different study that performed a genome-wide CRISPR-Cas9 knockout screen, identified that loss of *ARH3* or *PARP1* was associated with olaparib resistance, as well as with reduced basal LC3 flux, in LNCaP and C4 cells (Ipsen et al., 2022). Moreover, in *ARH3* or *PARP1* knockout cells, rapamycin exacerbated the detrimental effects of olaparib on C4 cell viability, whilst CQ did the opposite. Those results suggested a pro-cytostatic instead of cytoprotective role of autophagy in olaparib-treated prostate cancer cells, albeit validations with more specific modulation of autophagy were not performed. In summary, additional studies, which should include functional autophagy assays and additional ways to activate and inhibit autophagy (by targeting several different ATGs), are required to delineate and further clarify the role of autophagy in the regulation of prostate cancer cells’ sensitivity to olaparib. The potential influence of autophagy on the effects of other PARP inhibitors and Lu-PSMA also remains to be explored.

Among emerging drug-based targeted therapies that are not yet approved for clinical use in prostate cancer patients, the AKT inhibitor AZD5363 (capivasertib) is one of the most promising. AZD5363 was recently FDA-approved for use in combination with fulvestrant in a subset of breast cancer patients with one or more alterations in *PIK3CA*/*AKT1*/*PTEN* (Shirley, 2024). Such alterations, and especially mutations in *PTEN*, which renders the PI3K-AKT pathway constitutively active, are frequently found in prostate cancer (Choudhury, 2022). Two phase III clinical trials are ongoing for the use of AZD5363 in combination with either docetaxel + prednisolone or abiraterone in prostate cancer patients (Shirley, 2024). AKT inhibitors are also being tested in combination with PARP inhibitors in clinical trials (Choudhury, 2022). It has been suggested that autophagy mediates cytoprotective effects to counteract AZD5363-induced cell death in prostate cancer cells (Lamoureux et al., 2013). In that study, AZD5363 inhibited AKT and downstream mTOR activity in PC3 cells, and increased LC3 flux as assessed by Western blotting in the presence of Baf A1 or CQ. The detrimental effect of AZD5363 on cell viability was exacerbated along with increased caspase activity in PC3 cells co-treated with AZD5363 and Baf A1, CQ or 3MA. Moreover, transfection with an *ATG3*- or an *ATG7*-targeting siRNA increased AZD5363-induced apoptosis and caspase activation. Lastly, the authors demonstrated that the growth of PC3 xenograft tumours in mice was strongly reduced upon CQ and AZD5363 co-treatment (Lamoureux et al., 2013). These results suggested that targeting autophagy may enhance the efficacy of AZD5363 in prostate cancer treatment. As a cautionary note, however, it was not assessed whether the knockdown of *ATG3* or *ATG7* inhibited autophagy (neither with marker-based nor with functional autophagy assays), and since only one siRNA oligo was used per target, it cannot be excluded that potential off-target effects could have influenced the results. Moreover, since very similar effects were observed with the combination of AZD5363 and CQ in DU145 xenografts (which are defective in ATG5-dependent autophagy) as in PC3 xenografts (Lamoureux et al., 2013), the growth-inhibitory effects of CQ under those conditions are, at least in part, likely to be unrelated to inhibition of autophagy.

## 7 Discussion—limitations of current studies, main challenges and solutions

As evident from the overview and critical examination of current studies above, the role that autophagy may play in prostate carcinogenesis and prostate therapeutics remains highly uncertain. The conclusions drawn in the different studies range from autophagy impeding to promoting prostate cancer formation and progression, as well as from contributing to counteracting the effects of various therapeutic treatments. The same applies to the autophagy in cancer field in general (i.e., across all cancer types). The reason for the apparent paradoxical/dichotomous function of autophagy in cancer is often ascribed to its role being highly context- and cancer type-specific. Whereas this indeed very likely explains some of the apparent contradictory findings, we believe that above all, the main reason lies in the highly underestimated and under-communicated limitations in the methodological approaches that are used in the vast majority of current studies. Two aspects are of particular importance:

(i) First of all, the field is seriously impeded by being extremely reliant on the use of ***autophagic markers*** to assess alterations in autophagic activity, with LC3 being the by far most used marker. The problem with this is, as explained in the paragraph on autophagy methods above, that autophagy markers are insufficient and unreliable when used alone (Klionsky et al., 2021). Moreover, in most studies, there is uncertainty as to whether, or to which degree, the various pharmacological or genetic interferences/modulations of autophagy that have been employed have actually had their intended or anticipated effects on autophagic activity. In sum, we believe that this has resulted in many apparent contradictory findings, and, overall, in a critical lack of knowledge on the function of autophagy in cancer and on how autophagic activity changes during carcinogenesis as well as during anti-cancer therapy and development of treatment resistance.
(ii) Secondly, progress in the field is critically impeded by a general underestimation of what is required to firmly demonstrate ***causal*** relations between autophagy modulation and cellular/phenotypic effects. Many studies solely used pharmacological means to infer causality, which is associated with a very high degree of uncertainty due to the plethora of non-specific effects that all autophagy-modulating drugs exert on cells. Therefore, in this review, the majority of studies that have been selected from the literature are the ones that use at least one type of genetic interference with autophagy to infer causal relations. However, the extent to which this is necessary in order to firmly prove causality should not be underestimated. The reason for this is firstly that, as opposed to what was assumed upon their initial identification in the 1990s, most, if not all, ATG proteins have non-autophagic cellular functions (Yousefi et al., 2006; Gan and Guan, 2008; Baisamy et al., 2009; Hanson et al., 2010; DeSelm et al., 2011; Chung et al., 2012; Lee et al., 2012; Velikkakath et al., 2012; Maskey et al., 2013; Elgendy et al., 2014; Rohatgi et al., 2015; Chen et al., 2016; Joo et al., 2016; Joshi et al., 2016; Kaizuka and Mizushima, 2016; Nunes et al., 2016; Yang et al., 2016; Guo et al., 2017; Ramkumar et al., 2017; Wang and Kundu, 2017; Cadwell and Debnath, 2018; Saleiro et al., 2018; Galluzzi and Green, 2019; Hu et al., 2020; Lindner et al., 2020; Wu et al., 2020; Fang et al., 2021; Li et al., 2021; Mailler et al., 2021; Nieto-Torres et al., 2021; Sun et al., 2021; Zhang et al., 2022a; Hamaoui and Subtil, 2022; Rajak et al., 2022; Chen et al., 2023; Deng et al., 2023; Liang et al., 2023; Wang et al., 2023; Tedesco et al., 2024; Tran et al., 2024; Yoon et al., 2024) that are likely to influence the cellular/phenotypic effects that are observed upon their knockdown, knockout or overexpression, and many of these functions affect pathways that are highly relevant to cancer. Therefore, in order to infer causality, the same cellular/phenotypic effect must be observed upon interference with a number of different ATGs. Alas, this is very rarely done. Moreover, as mentioned in point (i) above, it is very important to use appropriate autophagy assays to verify that the knockdown or knockout (or overexpression) in question has actually had the intended effect on reducing (or increasing) autophagic activity. It cannot be taken for granted, since there are many different ATGs on which cells may show differential reliance, and on which different cell types may show different dependencies on in relation to cellular autophagic capacity. For instance, even very efficient knockdown of *ATG5*, whose gene product is one of the most central and critical in macroautophagy, has been shown to be insufficient or inefficient in reducing LC3-II levels in several types of mammalian cells (Hosokawa et al., 2006; Shin et al., 2013; Domagala et al., 2018), whereas *ATG5* knockout leads to complete disappearance of LC3-II (Hosokawa et al., 2006; Domagala et al., 2018; Szalai et al., 2015), and lipidated GABARAPs (Szalai et al., 2015). Thus, the fraction of cellular ATG5 protein required to maintain its role in autophagy seems to be very small. The same may not be the case for non-autophagic functions of ATG5. Hence, the possibility exists that *ATG5* knockdown may abolish several of the non-autophagic effects of ATG5, while at the same time failing to affect autophagic activity. In the studies discussed in the current review, all, except one study, have maximally interfered with two different ATGs to infer causality. Whilst some studies have not assessed how that affected autophagy (and instead just assumed that autophagy was impaired), others have used autophagic markers, but none have used functional assays to assess the effect of ATG interference on autophagic activity. This is a very important point, since, as mentioned above, autophagic markers are unreliable when used alone. Another important point, which applies to the whole autophagy field, is that few if any studies justify their choice of *which* ATG(s) they interfere with to infer causality between autophagy and cellular effects/phenotypes. The studies on autophagy in prostate cancer that have been discussed in the current review range from interfering with *ULK1*, *ULK2*, *ATG3*, *ATG4B*, *ATG4D*, *ATG5*, *BECLIN1*, *ATG7*, *LC3A*, *ATG16L1*, *FIP200*, *TFEB*, *LAMP2A*, or *p62.* While it is good that many different ATGs or autophagy regulators have been targeted in these studies, the problem is that the various studies have chosen just one or two targets in a seemingly random way to infer causality. Hence, it becomes very difficult to compare the results, since different ATGs and autophagy regulators have different non-autophagic functions, which are also likely to be affected upon knockdown/knockout. Additionally, the importance of using more than one different siRNA oligo or guide RNA and/or rescue experiments to rule out off-target effects should not be underestimated, as most studies discussed in this review have not done so. Finally, attention should be given to the fact that the stability of several of the ATGs depend on one another. For instance, as has been demonstrated in prostate cancer cell lines, knockout of *ATG7* leads to a severe depletion of ATG5 and ATG12 protein levels (which is at least in part due to ATG7 being required for ATG5-ATG12 conjugation) and partial depletion of ATG16L1 protein expression, whilst knockout of *ATG5* leads to a severe depletion of ATG12 and ATG16L1 proteins (Wible et al., 2019). Knockout or knockdown of *ATG5* and *ATG7* are therefore likely to result in many of the same influences on non-autophagic cellular processes that are associated with changes in the expression levels of ATG5, ATG12 and ATG16L1. Hence, demonstration of similar phenotypic effects upon genetic interference with *ATG5* and *ATG7* is not very strong evidence that those effects are specifically caused by autophagy inhibition. When deciding upon which ATGs to target in order to infer causal relationships with autophagy, it is advisable to choose ATGs who display minimal co-dependencies.
  Another limitation in the field of autophagy in cancer relates to the fact that most studies so far have focussed on the role of cancer cell-intrinsic autophagy in carcinogenesis and cancer therapy. It will be important for future studies to also explore the role of autophagy in the crosstalk between different cells of the tumour microenvironment, e.g., in relation to cancer-associated fibroblasts and various cells of the immune compartment. At present, the existing studies on this topic in prostate cancer are too sparse for inclusion into this review.

Lastly, whilst studies in the field have predominantly aimed to understand the role of classical macroautophagy in cancer, an emerging theme is the roles played by non-canonical, alternative autophagy pathways, for instance secretory autophagy (Gonzalez et al., 2020). Further explorations of such pathways, but also of selective types of autophagy and the relatively understudied CMA and microautophagy pathways, will lead to a more complete understanding of the role of autophagy in prostate cancer.

A number of clinical trials involving drugs that may alter autophagic activity are ongoing on prostate cancer cohorts (www.clinicaltrials.gov). These include drugs that may either inhibit (such as hydroxychloroquine, HCQ) or activate autophagy (such as rapamycin and derivatives). A main challenge is that the pre-clinical research data on which the clinical trials are grounded are suffering from the aforementioned limitations in understanding the causalities between autophagy modulation and biological effects. Therefore, it is at present not feasible to predict whether and in which contexts one should aim to inhibit or activate autophagy in prostate cancer therapy. Alas, the results from completed clinical trials involving autophagy-modulating drugs in prostate cancer patients (such as HCQ, pantoprazole, everolimus, and rapamycin) have not led to clarification of this point, since they have not been successful (NCT00786682) (Lemos et al., 2024; Mehnert et al., 2019). Another challenge with the current clinical trials is that none of the existing autophagy-modulating drugs are specific and could exert anti-cancer effects in an autophagy-independent manner—as has been reported for HCQ in melanoma and breast cancer cells (Maycotte et al., 2012; Maes et al., 2014). Therefore, in addition to the requirement for a better basic understanding of the role of autophagy in prostate cancer biology and therapy, drugs that are more specific at inhibiting or activating autophagy are needed for future testing in the clinical setting. Finally, a remaining challenge is to identify reliable methods to determine autophagy levels in patient tissue (Humbert et al., 2020) to verify that the autophagy-modulating drugs have had their intended effects.

## 8 Conclusion

Overall, current studies indicate that the macroautophagic pathway may play an important role in regulating prostate carcinogenesis as well as the response and development of resistance to prostate cancer therapeutics. However, and largely due to insufficient methodological approaches, it is still highly uncertain whether autophagy predominantly plays a tumour -suppressor or -promoting role, as well as whether autophagy plays a therapy-promoting or -resistant role. To overcome this, a shift in the field to focus on utilising functional autophagy assays, rather than the current general bias on almost exclusively using marker-based assays, is required. Moreover, the approaches for elucidating causality between autophagy modulation and biological effects through pharmacological and genetic interference with various ATGs and other central autophagy-regulating genes, must be substantially improved. Finally, future focus on the role of autophagy in the crosstalk between cells of the tumour microenvironment, as well as the role of selective autophagy, CMA, microautophagy, and various non-canonical autophagy pathways will lead to a more comprehensive understanding on the role of autophagy in prostate cancer.
